# Performance of an in-house real-time polymerase chain reaction for identification of *Mycobacterium tuberculosis* isolates in laboratory routine diagnosis from a high burden setting

**DOI:** 10.1590/0074-02760160048

**Published:** 2016-09-01

**Authors:** Juliana Failde Gallo, Juliana Maira Watanabe Pinhata, Erica Chimara, Maria Gisele Gonçalves, Lucila Okuyama Fukasawa, Rosangela Siqueira de Oliveira

**Affiliations:** 1 Instituto Adolfo Lutz, Centro de Bacteriologia, Núcleo de Tuberculose e Micobacterioses, São Paulo, SP, Brasil; 2 Instituto Adolfo Lutz, Centro de Imunologia, Laboratório de Diagnóstico Molecular de Infecções Bacterianas, São Paulo, SP, Brasil

**Keywords:** molecular diagnostics, *MPT64* protein, Mycobacterium tuberculosis, real-time polymerase chain reaction, tuberculosis

## Abstract

Brazil is one of the high burden countries for tuberculosis, and a rapid diagnosis is essential for effective control of the disease. In the present study, an in-house real-time polymerase chain reaction (PCR) assay targeting the *mpt64* gene for identification of *Mycobacterium tuberculosis* complex isolates was evaluated under routine diagnosis conditions in a reference laboratory. From May 2011 to July 2012, 1,520 isolates of mycobacteria were prospectively submitted for phenotypic and/or PRA-*hsp*65 identification and to real-time PCR. The *mpt64* real-time PCR showed 99.7% sensitivity and 96% specificity and detected 79.4% of the cases missed by phenotypic and PRA-*hsp*65 identification. The in-house real-time PCR assay showed high sensitivity and specificity and was successfully implemented in the routine diagnosis of tuberculosis in a reference laboratory from a high burden setting.

In 2014, an estimated 9.6 million people developed tuberculosis (TB) worldwide, and 1.5 million people died from the disease (WHO 2015). Brazil is one of the 22 high TB burden countries, with more than 67,000 cases diagnosed in 2014, of which 23% were from the state of São Paulo (SP) (MS 2015).

Isolation of *Mycobacterium tuberculosis* complex (MTC) from clinical specimens is the gold standard for TB diagnosis. In pure culture, identification of mycobacteria can be performed by either phenotypic or molecular techniques (de Waard & Robledo 2007). Phenotypic identification requires at least 15 days, whereas molecular diagnostics can be performed within hours (Espy et al. 2006 , Pfyffer 2007).

Real-time polymerase chain reaction (PCR) has been used with high sensitivity and specificity for the rapid identification of MTC clinical isolates (Miller et al. 2002, Richardson et al. 2009, Hong et al. 2011). Several regions of the mycobacterial genome have been used as targets by molecular assays, *e.g.*, IS*6110*, 16S rRNA, *hsp*65, *rpo*B, *sda*A, *dev*R and *mpt64* (Eisenach et al. 1990, Manjunath et al. 1991, Kadival et al. 1996, Lachnik et al. 2002, Kim et al. 2004, Chakravorty et al. 2005, Negi et al. 2007, Hwang et al. 2013, Nimesh et al. 2013).

The *mpt64* gene (GenBank accession no. NC_000962) is found as a single copy exclusively in the genome of MTC species (Lee et al. 1994) and has been used for both pulmonary and extrapulmonary TB diagnosis (Tan et al. 1995, Martins et al. 2000, Takahashi & Nakayama 2006, Pinhata et al. 2015). This gene has been reported to be very specific and sensitive when compared with other targets (Nimesh et al. 2013).

The Adolfo Lutz Institute (IAL) is a reference laboratory for TB in SP that has a well-established network composed of 291 local laboratories. Smear microscopy and culture are performed by these local laboratories, which then send the mycobacterial isolates to the IAL.

Nearly 7,000 isolates of mycobacteria from the local laboratories are received by the IAL annually. These isolates are routinely identified by conventional phenotypic techniques and PRA-*hsp*65 , and/or submitted to phenotypic drug susceptibility testing (DST), an assay currently performed if the patient meets one of these following criteria: has a positive culture at the 2nd month of treatment, has had contact with multidrug-resistant TB, is immunosuppressed, has a history of previous treatment or belongs to a vulnerable population (*e.g.*, homeless, immigrant, indigenous, inpatient and prisoner populations) (MS 2011).

The GeneXpert MTB/RIF (Cepheid, Sunnyvale, USA), a commercially available molecular system that detects the MTC and resistance to rifampicin (RIF) directly from biological specimens, was implemented at 37 local laboratories in SP for nearly two years and is used only for the detection of new cases or in retreatment cases for resistance detection. However, as only 37 of the 291 local laboratories have implemented this system, the majority of the local laboratory network still uses smear microscopy and culture for TB diagnosis. Furthermore, the sensitivity of the GeneXpert assay is reduced for sputum samples with a low volume as well as for paucibacillary specimens, and culture remains the gold standard in such cases.

In SP, most TB diagnoses still rely on culture, and the mycobacterial isolates have to be rapidly and accurately identified by the reference laboratory to promote effective TB control. Thus, the aim of this study was to evaluate the performance of an in-house real-time PCR assay targeting the *mpt64* gene for rapid identification of MTC isolates under routine diagnosis conditions to incorporate this technique in a reference laboratory with a very high demand for exams.

## MATERIALS AND METHODS

*Optimization of the mpt64 real-time PCR assay* - The limit of detection (LOD) of the real-time PCR assay was assessed before its implementation in the diagnosis routine. The primer and probe sequences for detection of the *mpt64* gene were the same as described by Takahashi and Nakayama (2006), and the assay was performed as previously reported by these authors with modifications to the oligonucleotide concentrations used. Primer concentrations from 0.1 to 0.9 µM and probe concentrations from 0.1 to 0.2 µM were tested. The DNA from the H37Rv isolate of MTC (ATCC 27294) was extracted and purified with a Genomic DNA from Tissue kit (Macherey-Nagel, Düren, Germany) and diluted in ultra-pure water to a concentration of 10 ng/µL. The LOD was obtained by amplification of 10-fold dilutions of this purified DNA from 10 ng/µL to 1 fg/µL. DNA concentrations were measured with a spectrophotometer. The LOD was determined to be the dilution that yielded a cycle quantification (Cq) value of 31.

After determination of the LOD, the molecular assay was evaluated using 268 isolates of 29 *Mycobacterium* species and 274 isolates of 36 non-*Mycobacterium* species (Table I). The assays were performed in a final reaction volume of 25 µL and were performed using TaqMan® Universal Master Mix (Applied Biosystems, Foster City, CA) with 2 µL of the isolates’ DNA. Forward primer, reverse primer and probe for *mpt64* were used in concentrations of 0.3, 0.3 and 0.1 µM. All of the isolates were tested in duplicate, and in each reaction, two wells for the positive control (H37Rv) and four wells for negative controls without DNA (two for the Master Mix preparation step and two for the DNA addition step) were included. The reactions were performed using the Roche LightCycler 480 II system (Roche Diagnostics, Indianapolis, USA) with the following cycling parameters: 50ºC for 2 min and 95ºC for 10 min followed by 40 cycles of 95ºC for 15 s and 60ºC for 1 min. Positive results were defined as Cq values ≤ 31, according to the LOD of the *mpt64* gene. All of the inconclusive results and inconsistent replicates were repeated. When repeated assays again resulted in inconclusive results, the isolate was sent for phenotypic and PRA-*hsp*65 identification.

**TABLE I:** Results of *mpt64* real-time polymerase chain reaction for 268 *Mycobacterium* sp. isolates and 274 non-*Mycobacterium* isolates tested

Organism	Strains (nº)	Strains positive for *mpt64* (nº)
*Mycobacterium tuberculosis*	124	124
*M. bovis*	3	3

Total	127	127

*M. abscessus*	11	0
*M. asiaticum*	1	0
*M. avium*	22	0
*M. bohemicum*	1	0
*M. bolletti*	1	0
*M. brumae*	1	0
*M. celatum*	1	0
*M. chelonae*	3	0
*M. flavescens*	2	0
*M. fortuitum*	11	0
*M. gordonae*	23	0
*M. immunogenum*	1	0
*M. intracellulare*	17	0
*M. kansasii*	19	0
*M. lentiflavum*	4	0
*M. mucogenicum*	3	0
*M. nonchromogenicum*	1	0
*M. parascrofulaceum*	1	0
*M. peregrinum*	8	0
*M. phocaicum*	1	0
*M. rhodesiae*	1	0
*M. scrofulaceum*	2	0
*M. simiae*	1	0
*M. smegmatis*	1	0
*M. szulgai*	1	0
*M. terrae*	1	0
*M. triviale*	1	0
*M. xenopi*	1	0

Total	141	0

*Acinetobacter baumannii*	1	0
*Corynebacterium diphtheriae*	2	0
*C. ulcerans*	1	0
*Chlamydia pneumoniae*	1	0
*C. trachomatis*	1	0
*Coccidioides immitis*	1	0
*Cryptococcus sp*	1	0
*Enterobacter cloacae*	1	0
*Escherichia coli*	5	0
*Enterococcus faecalis*	2	0
*Haemophilus aegyptius*	1	0
*H. influenzae*	54	0
*H. parainfluenzae*	3	0
*Histoplasma capsulatum*	1	0
*Klebsiella pneumoniae*	1	0
*Listeria monocytogenes*	17	0
*Legionella sp*	1	0
*Moraxella catarrhalis*	3	0
*M. genitalium*	1	0
*Neisseria lactamica*	2	0
*N. meningitidis*	55	0
*N. sicca*	2	0
*N. subflava flava*	1	0
*N. subflava perflava*	1	0
*Nocardia asteroides*	1	0
*Paracoccidioides brasiliensis*	1	0
*Pneumocystis carinii*	1	0
*Pseudomonas aeruginosa*	3	0
*Salmonella* Brandenburg	1	0
*S.* Enteritidis	1	0
*S.* Typhimurium	1	0
*Streptococcus agalactiae*	1	0
*S. mitis*	1	0
*S. pneumoniae*	67	0
*S. pyogenes*	1	0
*S. viridans*	36	0

Total	274	0

*Implementation of the mpt64 real-time PCR assay in the TB diagnosis routine* - From May 2011 to July 2012, 1,520 primary isolates of mycobacteria in liquid or on solid media from SP, Brazil, were included in the study. First, a presumptive identification according to micro- and macroscopic characteristics of the isolates was performed to classify these samples as MTC or non-tuberculous mycobacteria (NTM) isolates as described elsewhere (Monteiro et al. 2003, Coelho et al. 2007, Simeão et al. 2009) (Figure).

Next, *mpt64* real-time PCR was performed in the following cases: (i) isolates presumptively identified as MTC without DST criteria; and (ii) isolates that underwent DST but exhibited growth on PNB and BHI agar suggestive of contamination by other bacteria/fungi (Figure).

Phenotypic and PRA-*hsp*65 identification was performed when (i) isolates underwent DST but exhibited growth only on PNB or growth on PNB and on BHI agar suggestive of NTM; or (ii) isolates were presumptively identified as NTM (these were not included in the study) (Figure).

Phenotypic tests included growth rate (fast or slow), growth in temperatures of 26ºC and 37ºC, observation of colony pigmentation, and growth in the presence of 500 μg/mL of p-nitrobenzoic acid (PNB), sodium nitrite and picric acid (MS 2008). The first reading was performed after one week of incubation, and the isolates that showed no growth were incubated for one more week. PRA-*hsp*65 identification was performed as described by Chimara et al. (2008).

For real-time PCR DNA extraction, lysates of mycobacteria were prepared as follows: for isolates on solid media, a loop-full of organisms was suspended in 500 μL of ultra-pure water; for isolates in liquid media, 500 µL of bacterial growth was transferred to a sterile screw-capped tube. The suspensions were boiled at 100ºC for 20 min and frozen at -20ºC for at least 18 h (Chimara et al. 2008).

Real-time PCR was performed as described above. Isolates with Cq values from 14-31 were considered positive for *mpt64*; Cq values of 0 or ≥ 40 indicated that the isolate was negative; and Cq values from 32-39 were considered inconclusive results.

*Analysis of results* - Sensitivity, specificity, positive predictive value (PPV), negative predictive value (NPV), and accuracy of the *mpt64* real-time PCR assay were calculated under 95% confidence intervals (CI) using Microsoft Excel 2003 (Drobatz 2009). This work was approved by the Scientific Technical Committee of the Adolfo Lutz Institute, São Paulo, Brazil (19797/2009). Ethical approval was not required, as only mycobacterial isolates were analysed.

## RESULTS

The *mpt64* real-time PCR assay showed an efficiency of 94.69% (slope = -3.456, coefficient of correlation = 0.944) and an LOD of 20 fg of MTC genomic DNA. All of the 127 MTC isolates used in the evaluation of this assay before its implementation in the TB diagnosis routine were positive for *mpt64*. All of the isolates from other mycobacterial species and other genera yielded negative results (Table I).

Among the 1,520 mycobacterial isolates analysed prospectively, 1,352 (88.9%) were identified as MTC by presumptive features or phenotypic techniques and PRA-*hsp*65, including contaminated and mixed cultures. The real-time PCR assay identified MTC in 1,384 (91%) of isolates analysed (Table II).

**TABLE II:** Results of identification and *mpt64* real-time polymerase chain reaction (PCR) for the 1,520 mycobacterial isolates analysed

Real-time PCR	Presumptive or phenotypic and PRA-*hsp*65 identification						Total

	MTC	MTC + NTM	MTC + CO	NTM	CO	NG/NA	
Positive	1,332	13	2	4	21	12	1,384
Negative	4	0	0	96	17	5	122
Inconclusive	1	0	0	6	5	2	14

Total	1,337	13	2	106	43	19	1,520

CO: contamination; MTC: *Mycobacterium tuberculosis* complex; NG/NA: no growth and no amplification on PRA-*hsp*65 identification; NTM: non-tuberculous mycobacteria.

There were 13 isolates identified as a mixture of MTC and NTM, all of which were positive for *mpt64.* Regarding six of these mixed cultures, PRA-*hsp*65 analysis identified the species as follows: two isolates as MTC + *M. intracellulare*; two isolates as MTC + *M. avium*; one isolate as MTC + *M. kansasii*; and one isolate as MTC + *M. abscessus* subsp. *abscessus.* The remaining seven isolates did not amplify on PRA-*hsp*65 and the phenotypic tests could not identify the NTM species.

Three of the four NTM isolates that were positive for *mpt64* were identified by the PRA-*hsp*65 assay as *M. chelonae*, *M. kansasii* and *M. avium/M. colombiense.* The remaining NTM isolate did not amplify on PRA and the phenotypic tests revealed that this sample was an achromogen rapid grower mycobacterium.

Fifty-five (3.6%) of the isolates that could not be identified by the conventional techniques (contamination or no growth/amplification on PRA-*hsp*65) had valid real-time PCR results and corresponded to 55 different patients. To evaluate the performance of the real-time PCR for the *mpt64* gene for these isolates, we accessed the TB Notification System (TB-WEB) of the Center of Epidemiological Surveillance of SP. If the patient’s TB status was annotated in the system, the sample was considered to be from a confirmed TB case. Among these 55 isolates without identification results, there were 34 confirmed TB cases, of which 27 (79.4%) were detected by the real-time PCR assay. Of the 21 non-TB cases, real-time PCR results were negative in 16 (76.2%).

Isolates with inconclusive real-time PCR results and isolates that presented contamination or no growth/amplification on PRA-*hsp*65 (n = 69) were excluded from the analysis of sensitivity and specificity of the real-time PCR assay. Of the 1,451 isolates included in this analysis, 1,351 (93.2%) were identified as MTC by phenotypic tests and/or PRA-*hsp*65. Real-time PCR results were positive for MTC in 1,347 of these isolates (sensitivity of 99.7%). Among the 100 (6.8%) isolates that were negative for MTC by the culture assay, 96 were also negative in real-time PCR assay (96% of specificity) (Table III).

**TABLE III:** Performance of the *mpt64* real-time polymerase chain reaction (PCR) assay based on valid culture identification results

	Culture (n = 1,451)							
		
Real-time PCR	Positive	Negative	Total	Sensitivity (95% CI)	Specificity (95% CI)	PPV (95% CI)	NPV (95% CI)	Accuracy
Positive	1,347	4^*^	1,351	99.7% (99.2-99.9)	96% (90.1-98.9)	99.7% (99.2-99.9)	96% (90.1-98.9)	99.4%
Negative	4	96	100					

NPV: negative predictive value; PPV: positive predictive value; *: all were NTM isolates: one *Mycobacterium avium/intracellulare*, one *M. kansasii*, one *M. chelonae* and one achromogen rapid grower.

## DISCUSSION

Rapid identification of mycobacterial clinical isolates is essential for TB control and management of TB cases. The Adolfo Lutz Institute must be able to provide results in a short period of time because it is the reference laboratory for the state of São Paulo that diagnoses the majority of TB cases every year in Brazil.


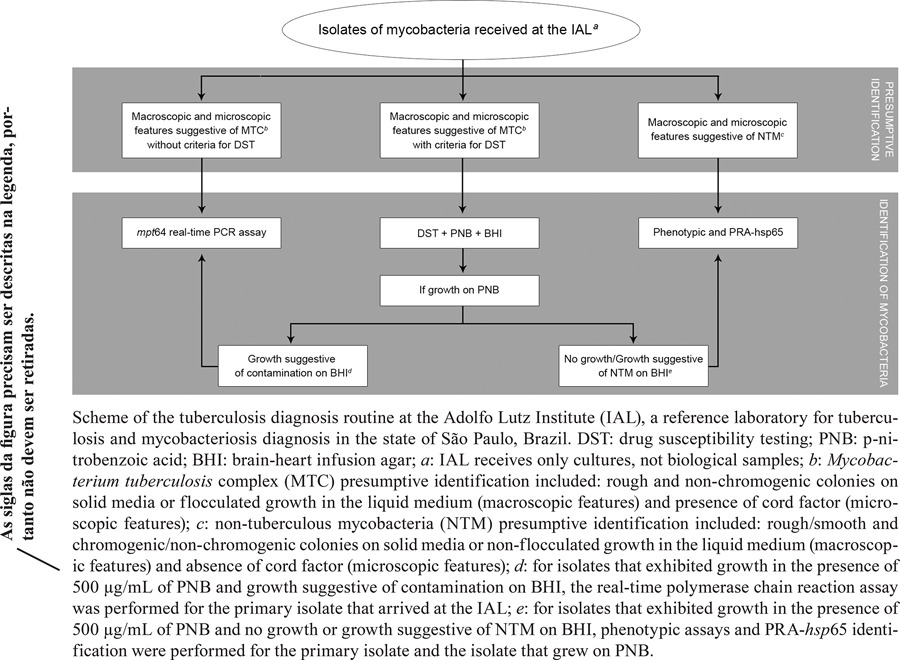


This is the first study that uses *mpt64* as a target to identify MTC primary clinical isolates. Detection of the *mpt64* gene was 100% sensitive and specific for MTC when real-time PCR was tested against previously identified mycobacterial and non-mycobacterial species. Under routine conditions, this assay also showed high sensitivity and specificity in the detection of MTC, as found by other authors (Gonçalves et al. 2012, Pinhata et al. 2015). Gonçalves et al. (2012) obtained high sensitivity and specificity using molecular beacons in an in-house real-time PCR assay to detect resistance to isoniazid and RIF in 988 MTC primary isolates received at the IAL. In this study, we decided to design a real-time PCR assay to identify MTC isolates in our TB diagnosis routine.

Pinhata et al. (2015) evaluated the performance of the *mpt64* real-time PCR assay directly in sputum samples and obtained the same sensitivity as that of culture (90.3%), as well as a specificity of 98.6%. Our study showed values of sensitivity and specificity of the real-time PCR assay above 96%. Moreover, this assay enabled the identification of MTC isolates in up to 48 hours, a substantial improvement in comparison to phenotypic methods, as phenotypic assays take at least two weeks to yield results.

In our TB diagnosis routine, results of identification by PRA-*hsp*65 are available in approximately four days, twice as long compared with real-time PCR. Furthermore, real-time PCR enables the parallel testing of 45 isolates at once, whereas in PRA-*hsp*65 identification, a maximum of 13 isolates can be included in an electrophoresis gel after enzymatic digestion. In PRA-*hsp*65 identification, a visual analysis of restriction profiles must be performed after the gel is run, whereas the real-time PCR system gives results that are already analysed.

Another great advantage of real-time PCR is that this assay detected 27/34 (79.4%) confirmed TB cases that were missed by phenotypic and PRA-*hsp*65 assay, allowing laboratory confirmation of TB.

The only inconclusive isolate in real-time PCR that was culture positive for MTC showed a Cq value of 33, a value very close to the LOD for the *mpt64* gene (Cq = 31), indicating that the amount of DNA in this culture was less than 20 fg. As this sample was a liquid culture, there could have been a very low quantity of bacilli, as the MGIT system is very sensitive in the detection of growth.

Four isolates positive for *mpt64* in the real-time PCR assay were identified as NTM. According to the TB-WEB system, two of these patients were being treated for TB at the study date because they presented clinical signs and symptoms, and both were cured. Between the other two patients with NTM, one had been previously treated for TB, whereas there is no annotated data regarding the other patient in the system. Presumably, cultures of these four patients were mixed MTC and NTM. However, as NTM grow faster, the amount of DNA isolated from these organisms was much higher for NTM than for MTC, and PRA-*hsp*65 identification detected only the NTM species. This can be explained by the fact that *hsp*65, used as a target on PRA identification, is encountered in all *Mycobacterium* species, whereas *mpt64* is specific for MTC.

In conclusion, this study demonstrates that the *mpt64* real-time PCR assay can be routinely used for rapid and accurate identification of MTC isolates from patients with no criteria for DST or in cases of mixed and contaminated cultures in laboratories with a high burden of cases and provided with infrastructure and personnel trained in molecular techniques.
